# How Do Therapists Experience 20‐Session Cognitive‐Behavioral Therapy for Anorexia Nervosa (CBT‐AN‐20)? A Qualitative Study

**DOI:** 10.1002/eat.70174

**Published:** 2026-07-28

**Authors:** Heather C. Duggan, Hannah M. Turner, Charlotte L. Rose, Jennifer J. Thomas, Kamryn T. Eddy, Kathryn G. Pasquariello, Kendra R. Becker, Debra L. Franko, Glenn Waller

**Affiliations:** ^1^ University of Sheffield, School of Psychology Sheffield UK; ^2^ Hampshire and Isle of Wight Health NHS Foundation Trust Southampton UK; ^3^ Avon and Wiltshire Mental Health Partnership NHS Trust Bristol UK; ^4^ Eating Disorders Clinical and Research Program, Massachusetts General Hospital Boston Massachusetts USA; ^5^ Department of Psychiatry Harvard Medical School Boston Massachusetts USA; ^6^ Northeastern University Boston Massachusetts USA; ^7^ Department of Psychology University of Warwick Coventry UK

**Keywords:** anorexia nervosa, CBT, clinicians, cognitive‐behavior therapy, eating disorders, evaluation, experiences, outpatient, qualitative, therapists' experiences

## Abstract

**Objective:**

We need to understand therapists' experiences of delivering manual‐based psychotherapies to identify possible dissemination barriers and opportunities. This study explored therapists' experiences of delivering CBT‐AN‐20, a new 20‐session cognitive‐behavioral intervention for outpatients with anorexia nervosa.

**Method:**

Ten therapists who had delivered CBT‐AN‐20 to at least one patient participated in an online survey. We used descriptive statistics (Likert‐scale questions; demographics) and thematic analysis (open‐ended text) to analyze responses. Pre‐registration: https://doi.org/10.17605/OSF.IO/R2U8P.

**Results:**

Therapists' mixed experiences of CBT‐AN‐20 were evident across quantitative and qualitative responses. Thematic analysis generated three main themes: Feelings about therapy over time; beliefs about for whom therapy might work; and perceptions of the characteristics of therapy. Despite some initial uncertainty, therapists reported both positive and negative experiences of delivering CBT‐AN‐20. They felt it worked well for some patients (highly motivated/less complex eating disorder presentations) but not for others (less motivated/more complex presentations). They experienced CBT‐AN‐20 as fast‐paced and action‐packed, yet with a clear, supportive structure. Therapists found encouraging rapid, early changes to patients' eating and weight difficult but worthwhile.

**Discussion:**

CBT‐AN‐20 is feasible and broadly acceptable for therapists to deliver. Therapists' mixed feelings were like those reported in qualitative studies of other psychotherapies for anorexia nervosa. While the manual addresses many identified challenges, training and supervision should focus on bolstering therapists' confidence in delivering CBT‐AN‐20 with fidelity. Future research should explore whether therapists' perceptions of CBT‐AN‐20 change with experience and if their expectations about whom CBT‐AN‐20 might work for are accurate.

## Summary

- This qualitative study explored therapists' experiences of delivering CBT‐AN‐20, a new 20‐session cognitive‐behavioral therapy for outpatients with anorexia nervosa.
- Results suggest that CBT‐AN‐20 is feasible and broadly acceptable for therapists to deliver.
- Therapists' training and supervision should address common challenges identified in this study.
- Future research should explore whether therapists' perceptions of CBT‐AN‐20 change with experience and if their expectations for whom CBT‐AN‐20 might work are accurate.

Cognitive‐behavioral therapy for eating disorders (CBT‐ED) has a strong evidence base (Agras and Bohon 2021; Waller and Beard 2024), supporting its widespread use in clinical practice. CBT‐ED is one of the recommended psychotherapies for adult outpatients with anorexia nervosa (American Psychiatric Association 2023; National Institute for Health and Care Excellence [NICE] 2017). However, even the best available treatments, like CBT‐ED, have limited patient outcomes in terms of recovery, remission, and relapse rates (de Rijk et al. 2024; Fichter et al. 2017; Khalsa et al. 2017). There are also typically long waiting lists before patients receive care (Austin et al. 2021; Beat 2021). Hence, National Institute for Health and Care Excellence [NICE] (2017) guidelines recommended exploring briefer yet effective therapies for eating disorders.

CBT‐AN‐20 is a new 20‐session evidence‐based CBT for outpatients with anorexia nervosa (Waller et al. 2026). Exploratory findings from a feasibility, acceptability, and preliminary effectiveness study of CBT‐AN‐20 (Duggan, Turner, et al. 2026) appear promising, with patients achieving similar outcomes (i.e., weight regain and reduced eating disorder symptoms) in 20 sessions to those of patients receiving 40‐session Enhanced CBT (CBT‐E; Fairburn 2008).

A study of patients' experiences of CBT‐AN‐20 (Duggan et al. 2025) informed the development of the manualized protocol (Waller et al. 2026). Results showed that patients valued the therapeutic relationship, expressed mixed opinions about therapy characteristics (i.e., structure and content), and experienced therapy differently over the course of treatment. Patients found the immediate emphasis on eating more for rapid weight regain difficult, leading to some dropouts. However, those patients who completed treatment reported that it was worthwhile in helping them move towards recovery. While informal feedback from therapists' training and supervision also informed the final protocol, no formal study of therapists' experiences of delivering CBT‐AN‐20 has been conducted.

Understanding therapists' feelings about and experiences of delivering manual‐based treatments is important for identifying potential barriers to effective delivery in clinical practice. Treatment fidelity impacts effectiveness (Peyser et al. 2021) and is influenced by therapists' expectations and experiences with change‐based therapies like CBT‐AN‐20. If therapists are uncomfortable with aspects of therapy, they may be less likely to deliver it accurately or at all (Waller 2009; Waller et al. 2012).

To our knowledge, no previous qualitative research has explored therapists' experiences of outpatient CBT for anorexia nervosa. Qualitative studies of other psychotherapies for anorexia nervosa (Easter and Tchanturia 2011; Rennick et al. 2024; Waterman‐Collins et al. 2014) and of CBT for non‐underweight cases (CBT‐T; Hewitt et al. 2025) highlight the challenges therapists face (e.g., confidence in treatment delivery, developing positive therapeutic relationships) as well as positive experiences of delivering these therapies (e.g., structure, protocol, flexibility).

In this study, we generated hypotheses about aspects of CBT‐AN‐20 requiring further development to ensure treatment fidelity, and identified positive experiences or aspects of treatment that might encourage its continued use by therapists in clinical practice. Therefore, we aimed to explore therapists' experiences of delivering CBT‐AN‐20, addressing the following research questions:

1. What are therapists' experiences of CBT‐AN‐20?

2. What do therapists' experiences tell us about how we might improve CBT‐AN‐20?

## 1 Method

### 1.1 Design

We followed our pre‐registered protocol (https://doi.org/10.17605/OSF.IO/R2U8P) and the Standards for Reporting Qualitative Research (SRQR; O'Brien et al. 2014). To facilitate comparisons between patients' and therapists' experiences of CBT‐AN‐20, we broadly replicated the cross‐sectional survey design used in our previous study of patients' experiences (Duggan et al. 2025).

### 1.2 Ethics

We obtained ethical permission from the University of Sheffield (ref. 066235) and the NHS (ref. 25/HRA/1419, IRAS 35620) to conduct this study. Participants gave informed consent for their data to be analyzed and included in published research (see Procedure).

### 1.3 Intervention: CBT‐AN‐20

CBT‐AN‐20 is a manualised evidence‐based 20‐session CBT for outpatients with anorexia nervosa (Waller et al. 2026). It has five phases. Phase 1 focuses on engagement, formulation, and active change. From Session 1 onwards, patients are exposed to the non‐negotiables of eating more and regaining weight at a rate of approximately 1 lb/0.5 kg per week or more. Patients are initially offered a limited number of sessions, with a review around Session 6. Treatment is drawn to a close if there is insufficient progress in weight restoration and changes to eating behaviors in Phase 1. Phase 2 introduces behavioral experiments designed to challenge patients' cognitions about food and weight. As patients are regaining weight and normalizing their eating behaviors, Phase 3 addresses any outstanding emotional issues. Phase 4 addresses body image concerns (e.g., using surveys and mirror exposure). Finally, Phase 5 involves planning for relapse prevention and ending treatment.

CBT‐AN‐20 is based on a similar therapeutic model to CBT‐T for non‐underweight eating disorders (Waller et al. 2019). Both focus on enhancing patient outcomes by encouraging early changes to eating behaviors (Chang et al. 2021; Vall and Wade 2015) and incorporate a review session to evaluate patients' progress. They use the inhibitory learning model of exposure (Craske et al. 2014), whereby patients are exposed to their maximally tolerable level of anxiety to fast‐track their learning by confronting their fears. CBT‐AN‐20 additionally addresses the specific needs of underweight patients (e.g., enhancing motivation and addressing the “anorexic voice” [Pugh and Waller 2017]). These features also distinguish CBT‐AN‐20 from existing CBT‐ED for anorexia nervosa (e.g., Fairburn 2008).

### 1.4 Participants

We invited 16 therapists from two National Health Service (NHS) outpatient eating disorder services and one charity who have delivered CBT‐AN‐20 to participate in this study. Therapists were eligible for inclusion if they had delivered CBT‐AN‐20 to at least one patient. The number of therapists completing the survey determined the sample size.

#### 1.4.1 Training and Supervision

All therapists attended a two‐day online training course led by a manual author (GW). Therapists had access to training materials to support them in delivering CBT‐AN‐20, but did not have the manual (Waller et al. 2026), which had not yet been published. Training materials consisted of a session‐by‐session treatment protocol, psychoeducational handouts, and training videos (https://sites.google.com/sheffield.ac.uk/cbt‐an‐20). The protocol formed the core of the manual, which includes minor adaptations (e.g., additional psychoeducational materials intended to enhance patients' meaningful engagement) based on informal and formal feedback (Duggan et al. 2025).

Clinical supervision was used to address therapists' questions and concerns about individual patients and therapy delivery, and to encourage treatment fidelity. Therapists received weekly small‐group (2–3) supervision with an experienced, qualified clinician. They presented each patient's case to the group at each meeting for supervisory guidance and peer comments. Feedback focused on evaluating patients' measurable progress (e.g., weight, psychometric scores), engagement, and level of risk, as well as discussing challenges in undertaking therapeutic tasks, planning for future sessions, and reflecting on therapists' experiences, including managing their anxieties.

### 1.5 Procedure

We designed an online Qualtrics survey (Data S1 and S2) with input from experienced therapists to ensure the questions were appropriately framed to capture therapists' experiences of delivering CBT‐AN‐20. This survey was cross‐sectional, being completed at a single time point. Participants were asked to retrospectively reflect on their thoughts and feelings about CBT‐AN‐20 before and after delivering therapy to capture their perceptions of changes over time. Eight Likert‐scale questions (e.g., “How willing did you think patients would be to engage in CBT‐AN‐20?”), required respondents to rate items on a scale from 0 (not at all) to 10 (completely). Nine questions (e.g., “Please describe your overall experience of delivering CBT‐AN‐20.”) required narrative responses. Qualitative survey questions could be skipped by entering “N/A”. Additionally, there were six demographic questions that therapists could opt to complete via a separate link at the end of the main survey. We made the demographics optional because the small number of eligible therapists increased the risk of individual identifiability. We stored demographic responses separately from those of the main survey to protect therapists' anonymity. Only one person (HD) had access to these potentially identifiable details, as she did not know any participants. All data were stored securely.

Data collection began in May 2025. NHS leads (HT and CR) and GW (for the charity) invited eligible therapists to participate via email (Data S3). Therapists were directed to the survey to read a detailed information sheet (Data S4) and give consent (Data S5), granting permission for the research team to analyze and publish their data (including anonymised quotes). Data collection ended in July 2025, when the research team agreed that extending the participation window was unlikely to generate further responses.

### 1.6 Data Analysis

We began the analysis during data collection and finalized it in April 2026. As in Duggan et al. (2025), we took a pragmatic approach to choosing appropriate methods of data analysis to address this study's research questions (Allemang et al. 2022; Feilzer 2010). We processed descriptive statistics and Cohen's Kappa in SPSS (v.29.0.2.0), and used Braun and Clarke's (2006) six‐step thematic analysis for the qualitative analyses (i.e., data familiarization, generating initial codes, generating themes, reviewing themes, defining themes, and report writing). We relied upon a single analyst (HD) for most of this process due to practical limitations.

HD conducted the familiarization and coding stages of the analysis during data collection. She added qualitative responses to a Microsoft Word transcript as they came in, and read them through at least twice for familiarization. HD coded the data (i.e., labeled segments of text to describe their meaning using comments), reviewing the codes at least twice. She then developed potential themes (i.e., shared patterns of meaning across codes) in Miro (an online whiteboard) by adding codes to virtual cards and grouping them into potential themes and subthemes (i.e., specific patterns of meaning situated within a broader theme). To avoid overrepresenting idiosyncratic perspectives in a small sample and to ensure representativeness across the dataset, we excluded subthemes with < 5 therapists (50% of the final sample) and themes with < 7 therapists (70%). The research team provided feedback on various iterations of the candidate thematic map.

HD worked with a second analyst (KP) to assess how well the fifth iteration of the thematic map represented the qualitative data. We replicated the same activities as in Duggan et al. (2025) (i.e., reading the transcript, reviewing the thematic map, and completing a task matching therapists' quotes to themes and subthemes), and calculated the level of agreement between analysts (see ORDA materials: https://doi.org/10.15131/shef.data.32076321). We used a random‐number selector (Haahr 2026) to choose three quotes per subtheme from all codes in the map for the matching task. We calculated percentage inter‐rater reliability (IRR) scores (Zhao et al. 2022) and Cohen's Kappa (Cohen 1960; McHugh 2012) for matching quotes to themes (83.3% agreement, Cohen's Kappa = 0.69) and subthemes (76.7% agreement, Cohen's Kappa = 0.74). Results indicated good agreement between raters (Laerd Statistics 2015). Subsequently, HD and KP discussed any discrepancies and KP's feedback on the thematic map. Both analysts agreed upon the final revised thematic map (Figure S1), giving 100% inter‐rater agreement.

### 1.7 Positionality and Reflexivity

While subjectivity is unavoidable in qualitative research, we included a second analyst to improve the credibility of this study's results by bringing an independent perspective that complemented and challenged the main analyst's interpretations of the data. We reflect upon the analysts' positionality in the Discussion.

## 2 Results

Ten therapists participated in this study (62.5% of the 16 invited).

### 2.1 Participant Characteristics

Eight (80%) therapists opted to provide demographic data, as shown in Table 1. They reported a mean of 1.9 (range: 0.8–4.5) years' experience in evidence‐based therapy for eating disorders.

**TABLE 1:** Demographic characteristics of therapists who delivered CBT‐AN‐20.

Demographics	*n* (%)
Sex at birth	
Female	8 (100.0%)
Age range (years)	
25–34	6 (75.0%)
35–44	1 (12.5%)
45–54	1 (12.5%)
Ethnicity	
White	6 (75.0%)
Southeast Asian	1 (12.5%)
Other: Chinese (East Asian)	1 (12.5%)
Highest educational level	
Masters	7 (87.5%)
Bachelor's degree	1 (12.5%)
Job title/position^a^	
Assistant psychologist	5 (62.5%)
CBT therapist	1 (12.5%)
Other: CBT‐T practitioner	1 (12.5%)
Other: Trainee clinical psychologist	1 (12.5%)

*Note:* *N* = 8. Percentages are reported to one decimal place.

Abbreviations: CBT, cognitive‐behavioral therapy; CBT‐T, 10‐session cognitive‐behavioral therapy for non‐underweight eating disorders (Waller et al. 2019).

^a^ These job titles/positions are UK‐specific and can be understood as follows. Assistant Psychologists are typically psychology graduates who are not yet qualified (licensed) clinical psychologists or therapists. However, they can deliver therapy, like CBT‐AN‐20, under the supervision of qualified professionals. CBT therapists are typically qualified professionals in their own right. CBT‐T practitioners may be either qualified therapists or Research Assistants/Assistant Psychologists delivering therapy under the supervision of qualified professionals. Trainee clinical psychologists are undergoing training to qualify as clinical psychologists, but are not yet qualified professionals, and deliver therapy under supervision.

### 2.2 Quantitative Analysis

Table 2 shows the therapists' quantitative survey responses, reflecting their feelings before and after delivering CBT‐AN‐20. The scores indicated mixed feelings about this therapy. Therapists rated their mean confidence levels, both in themselves and the treatment, higher after delivering CBT‐AN‐20 than before, though these changes were not significant (Table S1).

**TABLE 2:** Quantitative survey responses from therapists who delivered CBT‐AN‐20.

Survey questions^a^	*M*	Min.–Max. score
Before delivering therapy^b^		
How willing did you think patients would be to engage in CBT‐AN‐20?	4.70	3–6
How confident did you feel in the potential effectiveness of CBT‐AN‐20 for treating patients' eating disorders?	6.10	3–9
How confident did you feel in your ability to deliver CBT‐AN‐20 competently?	6.00	2–9
After delivering therapy^b^		
For patients who completed a full course of treatment (in either 20 sessions or faster), how effective was CBT‐AN‐20?	7.10	5–9
How confident did you feel in your ability to deliver CBT‐AN‐20 competently?	7.00	6–9
How likely is it that you would recommend CBT‐AN‐20 to other therapists?	7.70	5–10
How likely is it that you would recommend CBT‐AN‐20 to your loved one if they were to need treatment for anorexia nervosa?	7.20	4–10

*Note:* *N* = 10. CBT‐AN‐20 = 20‐session cognitive‐behavioral therapy for anorexia nervosa (Waller et al. 2026).

^a^ Responses were on a Likert scale from 0 = not at all to 10 = completely.

^b^ Therapists were asked to reflect on their thoughts and feelings before and after delivering therapy in this cross‐sectional survey to capture changes over time.

### 2.3 Qualitative Analysis

Figure S1 maps the results of the thematic analysis of therapists' qualitative survey responses, addressing Research Question 1. Three themes represent therapists' experiences of delivering CBT‐AN‐20: Feelings about therapy over time; beliefs about for whom therapy might work; and perceptions of the characteristics of therapy. Each includes responses from all 10 participants, demonstrating their representativeness across the dataset.

Ten subthemes capture specific elements of the broader themes. The number of therapists per subtheme (range: 7–10) indicates prevalence. The number of therapists represented by different perspectives is included as percentages of the total sample (*N* = 10). In some cases, these percentages exceed 100%, indicating that the same therapists could express different perspectives within the same subtheme. Table 3 presents anonymized quotes to illustrate each subtheme.

**TABLE 3:** Illustrative quotes by theme and subtheme.

Theme/subthemes	Nature of contribution	Illustrative quotes^a^
Theme 1: Feelings about therapy over time		
1.1 Feelings before delivering therapy	Unsure and apprehensive	*“I felt apprehensive as I had not worked with patients with anorexia nervosa before.”* Therapist 3
		*“I wasn't sure what to expect.”* Therapist 6
	Hopeful	*“I had had a very good experience delivering CBT‐10 [CBT‐T] so had high hopes for CBT‐AN‐20.”* Therapist 1
		*“Before delivering CBT‐AN‐20, I spent two years delivering CBT‐T, thus saw how effective CBT can be in the treatment of eating disorders. This gave me confidence in the effectiveness of CBT‐AN‐20 before delivering it.”* Therapist 2
	Negative expectations	*“[In CBT‐T] you can make a very strong case for making changes to eating as being a ‘medicine’ for stopping binge eating—which is extremely motivating for many patients. I did worry that before starting CBT‐AN‐20 I wouldn't have this sort of ‘leverage’ if the patient was not binge eating […]. This did make me wonder how engaged patients would be in the CBT‐AN‐20 therapy.”* Therapist 2
		*“I thought people would be less willing than with CBT‐T due to the early focus on change and weight monitoring.”* Therapist 4
1.2 Feelings after delivering therapy	Positive feelings	*“I found it very rewarding when clients did well with the model.”* Therapist 6
		*“I felt confident delivering it.”* Therapist 7
		*“I have had two patients who have done really well so far, and this has been positive.”* Therapist 9
	Negative feelings	*“It could feel like a constant compromise between protocol adherence and humanity for those who had difficulties with committing to non‐negotiables.”* Therapist 4
		*“I noticed myself feeling frustrated when clients were not making measurable progress—e.g.* *no increasing trend in weight.”* Therapist 10
	Mixed feelings	*“Overall, I have found [CBT‐AN‐20] to be a mixed bag. For some patients, it has been amazing, but for others, it has felt like I am asking too much of them too soon.”* Therapist 8
		*“It has been a mixed picture in terms of how successful this [therapy] has appeared to be*.” Therapist 9
Theme 2: Beliefs about for whom therapy might work		
2.1 Good for some patients	Lower risk	*“[CBT‐AN‐20 is most beneficial for] people who do not have a lot of weight to restore (perhaps BMI 17 or above). I think this treatment feels like an extra push for them on their [road to] recovery.”* Therapist 10
	Less complex presentations	*“[CBT‐AN‐20 is most beneficial for patients] who have fewer systemic factors that may impact recovery (so changes are reinforced rather than challenged/dismissed).”* Therapist 4
		*“I was unsure whether patients with less severe presentations or shorter time since onset would be easier to engage or more accepting of early weight gain.”* Therapist 9
	Prepared to engage	*“[CBT‐AN‐20 is most beneficial for] patients on board with expectations of making early changes to eating behaviors.”* Therapist 7
2.2 Not good for some patients	Longstanding disorders	*“I feel that this therapy may be less beneficial for those with longer‐standing eating disorders, who may be at a lower weight.”* Therapist 6
	Lack of illness severity	*“I think that for someone with less severe symptoms, this treatment relies on the patient being scared about it getting worse, and they might not be if they haven't experienced a more severe presentation.”* Therapist 9
	Ambivalence towards change	*“I have not found this treatment to be particularly helpful for patients who are ambivalent about change because there is so little focus on motivation.”* Therapist 8
2.3 Patients' motivation matters for recovery	Motivation matters	*“I think this therapy is most beneficial for patients who are already very motivated to change and are ‘up for giving it a go’*. Therapist 2
		*“Definitely people with anorexia nervosa who are not motivated or ready to begin [would benefit least from this therapy]*.” Therapist 5
		*“It was very rewarding to see clients doing well, and I feel that in 20 sessions, this is achievable if someone is motivated to change.”* Therapist 6
	Therapist suggestion	*“[Including] some sessions to allow for motivation.”* Therapist 8
Theme 3: Perceptions of the characteristics of therapy		
3.1 Fast paced and action packed	Fast paced	*“It is quite fast paced.”* Therapist 1
		*“The fast pace of the therapy was sometimes difficult as it placed high expectations on the patients. This sometimes made it a bit more difficult to manage risk in a community setting.”* Therapist 6
	Action packed	*“[I had] difficulties balancing this [family involvement and support needs] with the agenda and protocol.”* Therapist 3
		*“It is a lot in a short space of time.”* Therapist 4
		“*There is no time to unpack […] deep‐rooted issues that sustain anorexia nervosa as a coping mechanism*.” Therapist 10
	Therapists' suggestions	*“Simplify the agendas for the first couple of sessions. There is a huge amount to cover.”* Therapist 1
		*“A bit more time formulating difficulties and gaining a shared understanding of [some patients'] history, presentation and culture may be helpful.”* Therapist 3
		*“Streamlining the resources and session plans somehow?”* Therapist 9
3.2 Structure, support, and clarity matter	Structure matters	*“I, as someone who delivers the intervention, found the protocol for each session of CBT‐AN‐20 very helpful. It helps me keep track of what we need to and have covered in each session, and also of [the] overall progress that the patient makes.”* Therapist 5
		*“The structured nature with repeated psychometric measures, the use of different CBT techniques and various behavioral experiments really solidified my skills in delivering CBT*.” Therapist 10
	Support matters	*“I also found the regular supervision really helpful to problem solve any difficulties I was encountering with therapy.”* Therapist 6
	Clarity matters	*“There is a really clear process for addressing lack of change.”* Therapist 1
		*“I find the clear structure of [CBT‐AN‐20] helpful (food diaries, then weighing, then goals*, *etc.) as it sets clear expectations for patients (i.e.,* *“if I don't do the food diaries, then there will be nothing to discuss in session”). I find that this helps in being on the same page with the patient.”* Therapist 8
3.3. Weight regain is difficult, but change is worthwhile	Eating more and regaining weight is difficult	*“You are basically just trying to ask someone who is terrified of eating more to eat more!”* Therapist 2
		*“The speed at which weight gain was expected was challenging.”* Therapist 3
	Benefits of change	*“Just by regaining weight and starvation symptoms stopping means that my patient has much better cognitive flexibility, which in turn seemed to resolve the large majority of her body image issues without us even needing to use many of the body image techniques in CBT‐AN‐20.”* Therapist 2
		*“Early emphasis on non‐negotiables and change helped me as an early career therapist.”* Therapist 4
	Therapists' suggestions	*“[I] would've appreciated more guidance [on dietary planning for weight regain] or example meal plans.”* Therapist 3
		*“Perhaps knowing what to include or add to the patient's dietary structure and content for each week was a bit of a vague territory.”* Therapist 5
3.4 Nature of therapeutic relationships	Challenges to relationships	*“The relationship has become strained at times, particularly around the need for 0.5 kg a week weight gain, which some patients have complained feels too unrealistic to achieve, especially from week one*.” Therapist 8
		*“I was concerned about the relationship [as a result of applying firm empathy]. My main concern is someone thinking that I don't understand their difficulties, but I could work on better ways to communicate that.”* Therapist 9
	Positive relationships	*“I think my therapeutic relationship with my patients who have engaged have been good generally.”* Therapist 5
		*“Some really positive experiences and feedback from patients about [the] therapeutic relationship.”* Therapist 7
	Therapist suggestion	*“I wonder if there might be scope for a session around formulation at the start of the intervention, as this would be really beneficial in increasing understanding of the function of the [eating disorder], whilst also building on therapeutic rapport.”* Therapist 6
3.5 Comparisons with other therapies	Less favorable comparisons	*“I do think this therapy is a lot trickier than CBT‐T for ‘beginner’ therapists.”* Therapist 2
		*“I feel that these individuals [longer‐standing, low‐weight eating disorders] may benefit from CBT‐E with further emphasis on formulation, or MANTRA.”* Therapist 6
	Comparable with CBT‐T	*“The protocol was […] fairly similar to the CBT‐T protocol which I was familiar with.”* Therapist 3
		*“I had delivered CBT‐T previously and a lot of the techniques were the same, just with the added element of weight restoration*.” Therapist 8
	Favorable comparisons	*“I found it much clearer and easier to follow than CBT‐E.”* Therapist 1
		*“Better than guided self help for bulimia nervosa which is just surface level.”* Therapist 10

*Note:* Quotes use UK spellings and have been minimally edited for readability.

^a^ We have included the therapists' participant numbers to demonstrate that our example quotes are representative of responses across the entire dataset.

Finally, there were insufficient shared patterns of meaning in therapists' ideas for how we might improve CBT‐AN‐20 to include a specific theme or subtheme on this topic. Therefore, to address Research Question 2, therapists' suggestions for improvement are highlighted within each subtheme.

### 2.4 Theme 1: Feelings About Therapy Over Time

This theme explored therapists' reflections on their feelings about CBT‐AN‐20 over time. Its two subthemes highlight therapists' uncertainty before delivering therapy and how their feelings towards CBT‐AN‐20 changed after delivering it.

### 2.5 Subtheme 1: Feelings Before Delivering Therapy

Nine (90%) therapists reflected on their feelings about CBT‐AN‐20 before delivering it to patients. The most common feeling reported by eight (80%) therapists was uncertainty. Therapists were generally unsure about using this therapy, and some were apprehensive about working with patients with anorexia nervosa. However, four (40%) therapists felt hopeful about CBT‐AN‐20, with those who had previously delivered CBT‐T expressing positive views, given the outcomes they had experienced. Two (20%) therapists with experience delivering CBT‐T also shared negative expectations of CBT‐AN‐20, as they felt there might be additional motivational challenges to address when working with patients with anorexia nervosa, compared to non‐underweight eating disorders.

### 2.6 Subtheme 2: Feelings After Delivering Therapy

Therapists' reflections after delivering CBT‐AN‐20 expressed positive, negative, and mixed feelings. All 10 (100%) therapists provided positive feedback, reflecting their observations of its effectiveness for some patients and therapists' growth in confidence. However, six (60%) therapists also reported negative feelings, indicating that they experienced aspects of delivering CBT‐AN‐20 as challenging. Five (50%) comments directly reflected therapists' mixed experiences of CBT‐AN‐20, evident in the overall pattern of this subtheme.

### 2.7 Theme 2: Beliefs About for Whom Therapy Might Work

This theme explored therapists' beliefs about which patients might benefit from CBT‐AN‐20. Its three subthemes indicated that patients were perceived as better suited to this treatment if they had less complex eating disorder presentations and were motivated to change.

### 2.8 Subtheme 1: Good for Some Patients

Seven (70%) therapists suggested that treatment effectiveness was positively impacted by the nature of patients' eating disorders. CBT‐AN‐20 was seen as a helpful treatment for patients perceived by therapists as lower risk (e.g., higher baseline weight), or for those with less complex (e.g., fewer comorbidities and/or less challenging life circumstances) or severe (e.g., shorter illness duration) eating disorder presentations, as well as those prepared to engage in therapy.

### 2.9 Subtheme 2: Not Good for Some Patients

All 10 therapists shared opinions about which patients they felt CBT‐AN‐20 worked less well for. These opinions represent the inverse of those expressed in Subtheme 1. Seven (70%) therapists perceived patients with more complex comorbidities or severe eating disorder presentations as likely to find CBT‐AN‐20 more difficult. Three (30%) specifically highlighted patients with longstanding anorexia nervosa as likely to do poorly in this therapy. Conversely, one (10%) felt that a lack of illness severity might limit patients' motivation to change, suggesting that therapists did not always agree on for whom therapy worked less well. Lastly, five (50%) therapists noted that ambivalence towards change might also limit patients' outcomes.

### 2.10 Subtheme 3: Patients' Motivation Matters for Recovery

Seven (70%) therapists highlighted the importance of patients' attitudes on their outcomes in CBT‐AN‐20, suggesting that more motivated patients were likely to achieve better outcomes than less motivated or ambivalent patients. One (10%) therapist suggested adding sessions to specifically address motivation.

### 2.11 Theme 3: Perceptions of the Characteristics of Therapy

This theme explored therapists' perceptions of the characteristics of CBT‐AN‐20 (i.e., its structure and content). Its five subthemes reflected treatment aspects that therapists found challenging as well as those they found especially helpful.

### 2.12 Subtheme 1: Fast‐Paced and Action‐Packed

Eight (80%) therapists experienced the fast pace and action‐packed content of CBT‐AN‐20 as challenging. Three (30%) commented on its fast pace, meaning they had to work quickly to deliver its content. Six (60%) highlighted its action‐packed nature, meaning there was much content to cover in the available time, which was sometimes experienced as difficult. Taken together, delivering a lot of therapeutic content at a fast pace over a limited period contributed to a sense of CBT‐AN‐20 being time‐pressured. Resultantly, three (30%) therapists suggested reducing the content or increasing the available time to deliver it, particularly during early treatment.

### 2.13 Subtheme 2: Structure, Support, and Clarity Matter

Eight (80%) therapists valued the structure, support, and clarity built into the treatment protocol, training, and supervision. Seven (70%) commented on how they appreciated the structure of CBT‐AN‐20, meaning its overall shape and session‐by‐session protocol. Five (50%) highlighted that the clarity of both the protocol and the overall therapy structure was particularly helpful when working with patients. Three (30%) therapists also noted the importance of supervision—a key structural element—which supported them in delivering therapy effectively.

### 2.14 Subtheme 3: Weight Regain Is Difficult, but Change Is Worthwhile

Eight (80%) therapists found supporting patients through weight regain particularly difficult yet worthwhile. Seven (70%) reflected on how encouraging patients to consistently eat more and regain weight from Session 1 was difficult. However, five (50%) noticed the benefits of this approach to their patients' recovery (e.g., improved cognitive flexibility). Two (20%) therapists suggested a need for additional dietary guidance to improve participants' experiences of this challenging treatment stage.

### 2.15 Subtheme 4: Nature of Therapeutic Relationships

Nine (90%) therapists' experiences of relationships in CBT‐AN‐20 were shaped by its structure and content. Six (60%) experienced relational challenges due to tensions with patients regarding key aspects of this treatment (e.g., weight regain and firm empathy, meaning delivering treatment non‐negotiables with both fidelity and empathy). However, five (50%) reported positive experiences, suggesting that the characteristics of CBT‐AN‐20 did not necessarily impede the development of good relationships. One (10%) therapist suggested more time for formulation at the start of treatment to support the development of positive therapeutic relationships.

### 2.16 Subtheme 5: Comparisons With Other Therapies

Nine (90%) therapists shared mixed opinions about how CBT‐AN‐20 compared with CBT‐T (Waller et al. 2019), CBT‐E (Fairburn 2008), and guided self‐help. Five (50%) highlighted perceptions of CBT‐AN‐20's limitations, in terms of its difficulty for new therapists and appropriateness for patients with complex eating disorders. Three (30%) highlighted similarities between CBT‐AN‐20 and CBT‐T that they found helpful when delivering this therapy. Lastly, two (20%) therapists compared CBT‐AN‐20 favorably to other, more established therapies (i.e., CBT‐E and guided self‐help) in terms of its ease of delivery and depth of content.

## 3 Discussion

This study is the first to examine therapists' experiences of delivering CBT‐AN‐20. Furthermore, to our knowledge, it is the first study of therapists' experiences of outpatient CBT for anorexia nervosa. Thematic analysis generated three themes: Feelings about therapy over time, beliefs about for whom therapy might work, and perceptions of the characteristics of therapy. Taken together, therapists' experiences across these themes suggest that CBT‐AN‐20 was feasible and broadly acceptable to deliver. They reported that it worked well for some patients, supporting previous findings (Duggan et al. 2025). Therapists' suggestions and experiences may inform potential improvements to CBT‐AN‐20 and its delivery. While there is room for improvement to ensure treatment fidelity, results suggest that therapists find CBT‐AN‐20 a potentially useful treatment for some patients with anorexia nervosa.

The results of this study parallel therapists' mixed experiences of delivering other evidence‐based psychotherapies to patients with anorexia nervosa (Easter and Tchanturia 2011; Rennick et al. 2024; Waterman‐Collins et al. 2014). While therapists experience some aspects of delivering such therapies as challenging, other aspects support their effective delivery. For example, like CBT‐AN‐20 therapists in the present study, therapists who delivered Specialist Supportive Clinical Management in Waterman‐Collins et al.'s (2014) study found the focus on eating and weight difficult. In another example, like CBT‐AN‐20 therapists, those delivering Specialist Psychotherapy with Emotion for Anorexia in Kent and Sussex in Rennick et al.'s (2024) study valued regular supervision and training.

Our current findings are also consistent with our previous study of patients' experiences of CBT‐AN‐20 (Duggan et al. 2025). For example, both patients and therapists initially found CBT‐AN‐20's early push for weight regain and eating changes difficult. However, persisting with the treatment was deemed worthwhile in terms of patients' movement towards recovery.

The challenges of working with underweight patients in CBT‐AN‐20 (e.g., patients' lack of motivation or ambivalence about weight regain) appear to lead therapists to express hesitancy about delivering this therapy. Results reflected their uncertainty about encouraging patients to eat more and regain weight from Session 1. This hesitancy was evident when comparing CBT‐AN‐20 therapists' experiences to those of therapists delivering CBT‐T. Despite similarities in these therapeutic models (Waller et al. 2019, 2026), CBT‐T therapists were much more positive and confident working with non‐underweight patients (Hewitt et al. 2025) than our CBT‐AN‐20 therapists were working with underweight patients.

Therapists' hesitancy about delivering CBT‐AN‐20, and patients' ambivalence towards engaging with it, could reflect both patients' and therapists' fears about engaging in weight‐ and eating‐focused changes. For patients, a lack of motivation for recovery might arise from the ego‐syntonic nature of anorexia nervosa (Gregertsen et al. 2017), whereby aspects of the disorder (e.g., low weight and/or control over one's body) remain highly valued. For therapists, their hesitancy may reflect a lack of confidence or experience in delivering change‐focused therapies like CBT‐AN‐20 (Turner et al. 2014; Waller and Turner 2016). Alternatively, patients' perceived fragility (Meehl 1973) or therapists' anxieties about rupturing the therapeutic relationship (Mulkens et al. 2018; Waller et al. 2012) might lead them to avoid adopting the firm empathy approach, central to encouraging change in CBT‐AN‐20 (Waller et al. 2026). However, evidence shows that early symptom changes are stronger predictors of reduced eating disorder pathology than the alliance (Turner et al. 2015). Indeed, Graves et al.'s (2017) meta‐analysis showed that early symptom changes may improve the alliance. Ensuring that therapists understand the connection between change, alliance, and patient outcomes, and including this in supervision and training, is therefore an important factor in encouraging them to deliver CBT‐AN‐20 with fidelity.

Another possible explanation for therapists' hesitancy about delivering this therapy might relate to their uncertainty about for whom CBT‐AN‐20 might work. Our results suggest that therapists believe that CBT‐AN‐20 works well for motivated patients with less complex eating disorder presentations. However, to date, there is no evidence that these characteristics predict better treatment outcomes. Indeed, some studies (Duggan, Hardy, and Waller 2026; Eddy et al. 2017) have shown that patients can do well regardless of factors such as illness duration or severity. It is therefore important that we empirically explore for whom CBT‐AN‐20 works, and for whom it does not, so that therapists can have confidence in delivering this treatment and services can target their treatment offerings appropriately.

Some therapists' limited experience in treating underweight eating disorders might also explain their hesitancy about delivering CBT‐AN‐20 and their interpretation of patients' ambivalence towards it. While we do not know the specific details of participants' previous experience of working with anorexia nervosa, their qualitative responses suggested that, for some, delivering CBT‐AN‐20 was their first experience of working with this patient group. Therapists' previous experience is important because it can shape their feelings towards and responses to patients and therapeutic models. Less experienced therapists tend to report more negative feelings towards eating disorder patients (Thompson‐Brenner et al. 2012), more biased preconceptions of anorexia nervosa (Tragantzopoulou and Giannouli 2023), and more anxiety about delivering structured therapies (Turner et al. 2014) than experienced therapists. Therefore, we would need to know more about therapists' experiences of working with anorexia nervosa to more accurately contextualize our results.

Lastly, it is important to reflect on the positionalities of our analysts, which inevitably shaped their interpretations of our results. HD and KP both work with authors of the CBT‐AN‐20 manual. However, neither analyst was involved in CBT‐AN‐20's design, or in training or supervising therapists. HD (F, 42, White, UK) is a PhD candidate supervised by one of the manual authors (GW), with knowledge of CBT‐AN‐20 and previous experience in conducting thematic analyses. She has lived experience of an eating disorder, but no experience of delivering therapy. Having analyzed our patient survey data (Duggan et al. 2025), HD expected to find some similar themes in the current study. Recognizing this potential for bias in advance, and following our rules for excluding (sub)themes with too few therapists (see Data Analysis), ensured HD also looked out for contradictory (sub)themes. KP (F, 29, White, U.S.) is a PhD candidate in Clinical Psychology. She has experience delivering CBT‐AN‐20 and other therapies for eating disorders, which shaped her interpretations of the data and its analysis differently from HD. KP had no prior experience conducting thematic analyses and was not involved in any other aspect of this study.

### 3.1 Strengths and Limitations

This study's strengths lie in its contribution to knowledge about and development of CBT‐AN‐20. As the first study of therapists' experiences of CBT‐AN‐20 and, to our knowledge, of outpatient CBT for anorexia nervosa, it provides insights into how change‐focused therapies are experienced by those delivering them. Gathering such feedback from therapists at this early stage of intervention testing is crucial to making improvements, whether to the treatment protocol (e.g., the manual's focus on formulation‐building during the first session, based on informal feedback) or to therapist training (see Clinical Implications). Ensuring that CBT‐AN‐20 is acceptable to therapists so they are willing and able to deliver it effectively is key to maximizing patient outcomes (Waller 2009; Waller et al. 2012).

However, this study also has limitations. First, it had a small sample size, due to the limited pool of eligible therapists. Second, the sample itself was relatively homogeneous. Therapists were largely young White females with limited experience of delivering CBT‐AN‐20 and, in many cases, of delivering therapies for eating disorders more broadly. Some therapists' responses suggested that they had no prior experience of treating patients with anorexia nervosa at all. Results are thus not representative of more diverse and experienced therapists, limiting generalizability. Third, while therapists' qualitative survey responses implied heterogeneity in their prior training and experience of working with eating disorders, this study lacked clarity regarding participants' exact backgrounds, specifically in relation to treating anorexia nervosa. As a result, we recommend that our interpretations of therapists' responses (including the lack of representation of some participants' perspectives) in Theme 3, Subtheme 5 (Comparisons with Other Therapies) be considered cautiously. Fourth, this study relied predominantly on a single analyst, potentially limiting the reliability of the results. While not possible for this study, it would have been preferable to have two analysts involved in all stages of the data analysis. Fifth, we did not collect demographic data on therapists' gender identity; only on their sex at birth. Collecting such data in future studies would provide a more complete picture of participants' diversity. Sixth, this survey retrospectively asked about therapists' experiences at different time points (i.e., before and after delivering CBT‐AN‐20), potentially introducing recall bias and limiting the reliability and validity of our results. Lastly, therapists in this study had limited materials to support their delivery of CBT‐AN‐20, as the manual (Waller et al. 2026) had not yet been published. Using an earlier protocol might have affected patients' initial engagement in CBT‐AN‐20 because it did not include some of the psychoeducational materials later included in the manual, and subsequently may have affected therapists' perceptions of how well this therapy worked and for whom.

### 3.2 Clinical Implications

First, the manual (Waller et al. 2026) addresses many of the issues with CBT‐AN‐20 raised by therapists in this study (e.g., maximizing patients' motivation, more time for formulation, and clearer dietary guidance). Together with the web‐based materials (https://sites.google.com/sheffield.ac.uk/cbt‐an‐20/home), the manual should be used regularly by therapists to inform and develop their practice, and to troubleshoot issues as they arise. Second, training and supervision (see Method) should address the common challenges identified in this study to improve therapists' confidence in delivering this treatment with fidelity, thereby maximizing patient outcomes. These challenges include aspects of CBT‐AN‐20 that therapists found difficult (e.g., navigating treatment non‐negotiables and delivering a large amount of content within the available time), as well as misconceptions about anorexia nervosa (e.g., patients' perceived fragility [Meehl 1973] and illness duration limiting recovery potential [Eddy et al. 2017]). Third, CBT‐AN‐20 training and supervision may need further tailoring to meet therapists' needs. What is considered adequate may depend upon their prior training and experience (e.g., additional psychoeducation about working with anorexia nervosa for novice therapists). Therefore, to tailor training and supervision appropriately, we need to know more about therapists' specific concerns. Finally, it would be worthwhile to develop psychoeducational materials to train new therapists on the challenges and benefits of CBT‐AN‐20. Including quotes (e.g., Table 3) from therapists who have previously delivered CBT‐AN‐20 might validate new therapists' early experiences, support them in delivering treatment with fidelity, and encourage persistence with its more challenging aspects. We previously suggested a similar resource for CBT‐AN‐20 patients (Duggan et al. 2025).

### 3.3 Research Implications

To validate this study's findings, replication studies are needed with larger, more diverse samples of therapists, particularly those with more experience of delivering CBT‐AN‐20. Such studies will enable researchers to explore whether therapists' feelings about this therapy change as they become more confident in delivering it, and in working with anorexia nervosa more broadly. Replication studies are also needed to examine whether using the manual itself (Waller et al. 2026) improves therapists' experiences of treatment delivery and patient outcomes, given how the manual addresses many of the concerns raised in this study. Future studies should collect more detailed demographic data about therapists' prior training and clinical experience, including experience of and training in working with anorexia nervosa, other eating disorders, and mental health conditions. Including optional tick‐boxes (e.g., “lack of knowledge or experience”, “nothing further to add”) may allow for more accurate interpretations of participants' lack of responses than non‐responses (i.e., “N/A”). Collecting longitudinal data (e.g., therapists' confidence in CBT‐AN‐20 and their ability to deliver it) is also recommended to more accurately identify changes over the course of therapy. Finally, quantitative studies of patient outcome data, exploring potential moderating or mediating factors (e.g., patient characteristics, attitudes, and/or eating disorder presentations) are needed to determine whether data support therapists' beliefs about for whom CBT‐AN‐20 might work. Such knowledge should inform therapists' training and further refinement of the intervention, maximizing its effective delivery to patients.

## Author Contributions

**Kamryn T. Eddy:** methodology, funding acquisition, writing – review and editing, project administration. **Kendra R. Becker:** conceptualization, methodology, project administration, supervision, validation, writing – review and editing. **Heather C. Duggan:** conceptualization, investigation, writing – original draft, writing – review and editing, project administration, resources, methodology, data curation, formal analysis, validation, visualization, supervision. **Charlotte L. Rose:** methodology, project administration, writing – review and editing. **Glenn Waller:** conceptualization, writing – review and editing, methodology, validation, project administration, supervision, resources, visualization. **Jennifer J. Thomas:** methodology, project administration, funding acquisition, writing – review and editing. **Kathryn G. Pasquariello:** validation, writing – review and editing. **Debra L. Franko:** conceptualization, methodology, validation, writing – review and editing, project administration, supervision. **Hannah M. Turner:** methodology, project administration, writing – review and editing.

## Lived Experience Involvement Statement

A subset of the authors has lived experience of an eating disorder, including the lead author (H.D.). All authors other than the lead author have experience delivering therapy to patients with eating disorders.

## Funding

H.D. receives PhD funding from the White Rose (ESRC) Doctoral Training Partnership [grant number: ES/P000746/1] for her PhD. For this specific study, H.D. also received a stipend from the Rubenstein Charitable Foundation to participate in the Eating Disorders Summer Research Fellowship (Eating Disorders Clinical and Research Program, Massachusetts General Hospital/Harvard Medical School). J.J.T. receives funding from the National Institute of Mental Health (K24MH135189) to support her mentorship of early‐career investigators in the field of eating disorders. The funders had no role in the conceptualization of this study, the study design, data collection, data analysis or interpretation of the data, writing the manuscript, or the decision to submit the paper for publication.

## Disclosure

We completed the Original Article checklist as described in the author guidelines.

## Ethics Statement

Ethical permission to conduct this study was obtained from the University of Sheffield (ref. 066235) and the National Health Service (NHS; ref. 25/HRA/1419, IRAS 35620).

## Conflicts of Interest

Five of the authors (C.L.R., H.M.T., J.J.T., K.T.E., and G.W.) receive royalties from Routledge for the CBT‐AN‐20 manual (Waller et al. 2026). The other authors of this study have no conflicts of interest to declare.

## Supporting information


**Data S1:** eat70174‐sup‐0001‐Supinfo1.docx.

**Data S2:** eat70174‐sup‐0002‐Supinfo2.docx.

**Data S3:** eat70174‐sup‐0003‐Supinfo3.docx.

**Data S4:** eat70174‐sup‐0004‐Supinfo4.docx.

**Data S5:** eat70174‐sup‐0005‐Supinfo5.docx.

**Figure S1:** Thematic map of therapists' qualitative survey responses.

**Table S1:** Comparison of therapists' estimates of their confidence in treatment effectiveness and their ability before and after delivering CBT‐AN‐20.

## Data Availability

Materials are currently available for reviewers/editors to view at the following private link: https://figshare.com/s/0a1253758711502b21ff. Data supporting the results of this study will be made openly available at the following link: 10.15131/shef.data.32076321.
