# Supplementary material for: How Do Therapists Experience 20‐Session Cognitive‐Behavioral Therapy for Anorexia Nervosa (CBT‐AN‐20)? A Qualitative Study
Source: Int J Eat Disord. 2026 Jul 28;59(11):2508–20. doi: 10.1002/eat.70174 (PMC13726451; doi:10.1002/eat.70174)
Supplement: Supplementary file 1 [file EAT-59-2508-s001.docx]

**Consent Form Text for CBT-AN-20 Therapist Survey**

**Consent Form**

**Study Title:** How Do Therapists Experience 20-Session Cognitive-Behavioural Therapy for Anorexia Nervosa (CBT-AN-20)?

Please read the following information carefully and tick the appropriate boxes.

**Taking part in the project**

I have read and understood the project information sheet and have downloaded a copy for my own records. (If you answer ‘No’ to this question, please do not proceed with the consent form until you are fully aware of what taking part in this project will mean).

Yes ☐ No ☐

I have been given the opportunity to ask questions about this project.

Yes ☐ No ☐

I understand how to raise a concern or make a complaint.

Yes ☐ No ☐

I agree to take part in this project. I understand that taking part in this project will include completing an online Qualtrics survey about my experiences of delivering CBT-AN-20.

Yes ☐ No ☐

I understand that choosing to participate as a volunteer in this research does not create a legally binding agreement, nor is it intended to create an employment relationship, with the University of Sheffield.

Yes ☐ No ☐

I understand that I can withdraw my data from this study at any point during data collection. If I choose to withdraw my data, I understand it may not be possible to remove the research data I have contributed one month after data collection starts [exact date to be added here], after which the data will be in the process of being analysed.

Yes ☐ No ☐

I understand that I can withdraw my data from being published as Open Data at any point up to 1 August 2025, after which anonymised data will be uploaded to an open-access repository.

Yes ☐ No ☐

**How my information will be used during and after the project**

I understand my personal details will not be revealed to people outside the project.

Yes ☐ No ☐

I understand and agree that my words may be quoted in publications, reports, web pages, and other research outputs.

Yes ☐ No ☐

I understand and agree that other authorised researchers will have access to this data only if they agree to preserve the confidentiality of the information as requested in this form.

Yes ☐ No ☐

I understand and agree that other authorised researchers may use my data in publications, reports, web pages, and other research outputs, only if they agree to preserve the confidentiality of the information as requested in this form.

Yes ☐ No ☐

I give permission for the anonymous survey data that I provide to be deposited in ORDA, the University of Sheffield data repository.

Yes ☐ No ☐

So that the information you provide can be used legally by the researchers:

I agree to assign the copyright I hold in any materials generated as part of this project to the University of Sheffield.

Yes ☐ No ☐

**Create a participant ID number**

Please now create a memorable ID containing the first two letters of your mothers’ maiden (pre-married) name, the number of the month of your mother’s birth, and the last two letters of your mother’s given name (e.g., SMI3NE). If you do not have these details, please choose another important person in your life whose details you can recall (e.g., father, partner) and create your ID in the same way. This will be your participant ID. You will need to keep this ID in case you need to correspond with the research team in future, as we will not have other identifying details (e.g., your name, email address). Your ID will not be used in any research publication. Participants will be given a number in any research outputs (e.g., “Participant 1”), including in any Open Data.

**Informed consent for participation in this study**

In lieu of my signature (to protect my anonymity), by ticking the following box, entering my participant ID, and the date, I fully consent to participate in this project.

**I provide my fully informed consent to participate in this project.** ☐

**Participant ID** *(please enter the memorable ID you have created in the previous step)* ______

**Date** DD/MM/YYYY

**Email Updates**

Please provide an email address below if you would like to be informed of any publications arising from this study.

There is no obligation to provide an email address. Email addresses will only be used to notify participants of the publications arising from this study. They will not be used for any other purpose. Email addresses will be stored separately from all other survey information so that it cannot be linked to your responses and will be deleted three months after Heather Duggan’s PhD thesis is published.

Email: _________

**Project contact details for further information**

Lead researcher: Heather Duggan ([hduggan1@sheffield.ac.uk](mailto:hduggan1@sheffield.ac.uk)).

Supervisor: Prof. Glenn Waller ([g.waller@sheffield.ac.uk](mailto:g.waller@sheffield.ac.uk)).

Head of Department: Dr Chris Martin ([psy-hod@sheffield.ac.uk](mailto:psy-hod@sheffield.ac.uk)).
