# Supplementary material for: How Do Therapists Experience 20‐Session Cognitive‐Behavioral Therapy for Anorexia Nervosa (CBT‐AN‐20)? A Qualitative Study
Source: Int J Eat Disord. 2026 Jul 28;59(11):2508–20. doi: 10.1002/eat.70174 (PMC13726451; doi:10.1002/eat.70174)
Supplement: Supplementary file 2 [file EAT-59-2508-s002.docx]

**Information Sheet Text for CBT-AN-20 Therapist Survey**

**Important Information: Please read before consenting to participate in this study.**

**Study Title:** How Do Therapists Experience 20-Session Cognitive-Behavioural Therapy for Anorexia Nervosa (CBT-AN-20)?

**Research Team**

The lead researcher for this study is Heather Duggan, a PhD researcher from the School of Psychology at the University of Sheffield, under the supervision of Glenn Waller, in collaboration with clinical leads in Boston, Bristol, Sheffield, and Southampton where therapists have been delivering CBT-AN-20.

**Invitation**

You are invited to participate in the above-titled research study exploring therapists’ experiences of delivering CBT-AN-20, a novel evidence-based therapy for patients with anorexia nervosa. Your responses at this early stage of intervention development will help to shape future iterations of this novel therapy.

Before you decide to take part, it is important for you to understand why the research is being done and what it will involve. Read the following information carefully and discuss it with others (or contact the research team using their details below) if there is anything you do not understand or if you would like more information before deciding whether to take part.

**What is the study’s purpose?**

This study is a survey that aims to explore therapists’ experiences of CBT-AN-20, a novel evidence-based therapy for patients with anorexia nervosa. It will address a gap noted in a previous study of patients’ experiences (Duggan et al., 2024, manuscript in preparation) by providing a more holistic understanding of participants’ experiences – both therapists and patients. Results will help researchers understand how best to shape future iterations of this therapy to improve patient outcomes**.**

Therapists who have delivered CBT-AN-20 are invited to complete an online Qualtrics survey in Spring 2025. Results will be analysed and written up for publication in a peer-reviewed journal by Autumn 2025 and will be included in the lead researcher’s PhD thesis. When providing consent, you will be given the option to provide an email address to be informed of any future publications.

**Why have I been chosen?**

Therapists who have delivered CBT-AN-20 as either part of the feasibility and acceptability study or in preparation for an upcoming effectiveness study are invited to share their experiences of this new therapy by completing this survey.

**Do I have to take part?**

It is entirely up to you to decide whether to take part in this study. If you do decide to take part, you can download this information sheet to keep and you will be asked to sign a consent form. You can withdraw from this study at any time during data collection up to one month after data collection starts [exact date to be added here]. After this date, the analysis will have started, and you can no longer withdraw your data. Taking part in this research is entirely voluntary and will not create any legally binding agreement or any form of employment with the University of Sheffield.

**What will happen to me if I take part? What do I have to do?**

If you choose to participate in this voluntary study, you will complete a consent form and an online survey via Qualtrics (the platform where you are currently reading this information). The survey comprises three sections and takes up to 20 minutes to complete. Section A asks about your expectations of CBT-AN-20 before starting to deliver treatment. Section B asks about your experiences of delivering CBT-AN-20. Section C asks for demographic information. The survey contains a mix of open-ended and closed questions. Please complete as many questions as you feel comfortable and provide as much detail as possible. Once you have pressed the button on the final page to submit your responses, you will have finished the survey.

**What are the possible disadvantages and risks of taking part?**

There are no anticipated disadvantages, discomforts, or risks of participating in this study, as evaluating new therapies is part of the everyday clinical role of therapists. The clinical leads at the participating services support participation in this study. However, choosing not to participate will have no adverse impacts. If there are any unanticipated negative impacts of taking part, therapists are advised to speak about these in supervision or raise a complaint with the research team as described below.

**What are the possible benefits of taking part?**

By taking part in this study, you will be contributing to our knowledge of CBT-AN-20 during the early stages of development of this novel intervention. Your experiences may help researchers improve training for therapists and shape future iterations of this therapy to improve patient outcomes.

**Will my taking part in this project be kept confidential?**

Yes. Your participation in this survey will remain confidential throughout this study. The survey will be anonymous. If you decide to participate in this study, you will be asked to create a memorable ID containing the first two letters of your mother’s maiden (pre-married) name, the number of the month of your mother’s birth, and the last two letters of your mother’s given name (e.g., SMI3NE). You will need to keep this ID in case you need to correspond with the research team in future, as we will not have your name or email address. If you opt to share your email address for updates about future publications, this will be stored separately from other survey data to protect your anonymity. Your ID will not be used in any research publication. Participants will remain anonymous in any publications and be given a number in any research outputs, including any Open Data.

All personal data will be kept strictly confidential. Your consent for participation and email address (if you choose to provide this) will be kept separately from your responses and stored in a password-protected folder that is only accessible to the Sheffield research team. Your demographic survey responses will also only be accessible to the Sheffield research team. Anonymised responses to the survey questions (Sections A and B) will be shared with Boston researchers working with the Sheffield team on the data analysis. Your participant ID will remain confidential with the Sheffield research team. Your anonymity will also be protected in any research outputs, including Open Data.

**Data Protection**

The University of Sheffield will act as the Data Controller for this study. This means the University is responsible for looking after your information and using it properly. The Sheffield research team will act as the main Data Processors for this study. This means that they will be responsible for downloading your anonymised data from Qualtrics and storing this information securely. Your consent for participation (and email address, if provided) will be stored separately from your survey responses in a password-protected folder only accessible by the Sheffield research team. Anonymous responses to the survey questions (excluding demographics) will be shared with the Boston researchers involved in the data analysis.

Anonymised data will be stored indefinitely as Open Data. Open Data is part of the Open Science movement and refers to data that has been made freely available to be downloaded and used by others. Making data Open Access is important because it allows us to be transparent as researchers, sharing our data with others for scrutiny, and also enables other researchers to explore the answers to new questions. If you do not wish for your anonymised responses to be included as Open Data, you can indicate this on the consent form and can withdraw permission for this up until 1 August 2025, after which anonymised data will be made Open Access.

This research is carried out in the public interest in accordance with data protection legislation. Further information can be found in the [University’s Privacy Notice](https://www.sheffield.ac.uk/govern/data-protection/privacy/general). Some of your data collected during the research is defined in the legislation as more sensitive (e.g., ethnicity), so we need to let you know that the use of this data is necessary for archiving purposes in the public interest, scientific research purposes, or statistical purposes.

**What happens to the results of the research?**

The results of this study will be written up for publication in a peer-reviewed journal and the lead researcher’s PhD thesis. Participants will be anonymous and referred to using a number (e.g. ‘Participant 1’) in all publications. A copy of the PhD thesis will be stored in the White Rose repository upon completion (https://eprints.whiterose.ac.uk/) and will contain links to published papers. Participants can choose to provide their email address to be notified of any future publications. You are also welcome to contact the lead researcher at any point if you would like further details of any publications.

Anonymised data from this study will be published Open Access via the University of Sheffield’s ORDA system (https://orda.shef.ac.uk/). Due to the nature of this research, it is likely that others may find the data collected useful in addressing interesting and important questions in the future. We will ask for your explicit consent for your data to be shared. You can withdraw consent for sharing your data as Open Data before 1 August 2025, at which point data will be shared with publishers as part of the process of submitting a manuscript for publication.

**Who is organising and funding the research?**

The lead researcher is conducting this study as part of a PhD funded by the ESRC White Rose Doctoral Training Partnership (grant number: ES/P000746/1). The funder is not involved in the design, conduct, and publication of this research.

**Who has ethically reviewed the project?**

This project has been ethically approved by the School of Psychology at the University of Sheffield [INSERT ETHICS APPICATION NUMBER].

**What if something goes wrong, and I wish to complain about the research or report a concern or incident?**

If you would like to complain about any aspect of the research or if you feel that you have been exploited, abused, or harmed as a result of taking part in this research, please contact Glenn Waller ([g.waller@sheffield.ac.uk](mailto:g.waller@sheffield.ac.uk)) in the first instance. If you feel your complaint has not been handled in a satisfactory way, you can contact the Head of the School of Psychology, Dr Chris Martin (psy-hod@sheffield.ac.uk). If your complaint is about how your personal data has been handled, you can find information on how to raise a complaint in the [University’s Privacy Notice.](https://www.sheffield.ac.uk/govern/data-protection/privacy/general)

**Contacts for further information**

Lead researcher: Heather Duggan ([hduggan1@sheffield.ac.uk](mailto:hduggan1@sheffield.ac.uk)).

Supervisor: Prof. Glenn Waller ([g.waller@sheffield.ac.uk](mailto:g.waller@sheffield.ac.uk)).

*Please download and keep a copy of this information sheet for your records. If you choose to participate in this study, we also advise you to take note of your participant ID. You could save this document with your ID in the header (e.g. SMI3NE_Information Sheet_DATE) to ensure you have a record of this number.*

**Thank you for reading this information and we hope you will take part in this study.**
