# Supplementary material for: How Do Therapists Experience 20‐Session Cognitive‐Behavioral Therapy for Anorexia Nervosa (CBT‐AN‐20)? A Qualitative Study
Source: Int J Eat Disord. 2026 Jul 28;59(11):2508–20. doi: 10.1002/eat.70174 (PMC13726451; doi:10.1002/eat.70174)
Supplement: Supplementary file 3 [file EAT-59-2508-s003.docx]

**Supporting Information F**

**Figure F1**

*Thematic Map of Therapists’ Qualitative Survey Responses*

*Note. N* = 10.
