# Supplementary material for: How Do Therapists Experience 20‐Session Cognitive‐Behavioral Therapy for Anorexia Nervosa (CBT‐AN‐20)? A Qualitative Study
Source: Int J Eat Disord. 2026 Jul 28;59(11):2508–20. doi: 10.1002/eat.70174 (PMC13726451; doi:10.1002/eat.70174)
Supplement: Supplementary file 4 [file EAT-59-2508-s004.docx]

**Additional Qualtrics Text for CBT-AN-20 Therapist Survey**

This document contains additional Qualtrics text for use alongside the information sheet text, consent form text, and survey questions to create the completed survey.

**Survey Structure**

The first page will contain an overview of the study and what participation will involve. The second page will contain the information sheet and consent form. Once participants’ consent is obtained, the survey questions will be presented, with each section starting on a new page, or over several pages for longer sections to break up the questions and make them easier to read (see document “Therapist Survey Questions”). The final section of survey questions (Section C “About you”) will be created as an embedded survey within the broader Qualtrics survey, keeping participants’ demographics separate from their main survey responses for confidentiality purposes. The last page of the survey will reiterate key information, allow participants to submit their responses, and thank them for their participation.

**Page 1:**

**About this Survey**

Thank you for your interest in completing this survey. You are invited to participate in a research study titled **‘How Do Therapists Experience 20-Session Cognitive-Behavioural Therapy for Anorexia Nervosa (CBT-AN-20)?’** Your responses at this early stage of intervention development will help to shape future iterations of this novel therapy.

This study is being done by Heather Duggan from the School of Psychology at the University of Sheffield, under the supervision of Glenn Waller, in collaboration with clinical leads in Boston, Bristol, Sheffield, and Southampton where therapists like you have been delivering CBT-AN-20.

The survey comprises three sections and will take up to 20 minutes to complete. Section A asks about your expectations of CBT-AN-20 before you started to deliver this treatment. Section B asks about your experiences of delivering CBT-AN-20. Section C asks demographic questions. The survey contains a mix of open-ended and closed questions. Please complete as many questions as you feel comfortable with and provide as much detail as possible.

If you are interested in taking part, please read the following information and complete the consent form. You will then be guided through each section of the survey.

[Next button]

**Page 2:**

**Study Information and Consent**

Please read the following information and download a copy to retain for your records before starting the survey.

[Text from information sheet]

[Link to downloadable information sheet]

Before submitting your survey, we need to record your consent to take part in this study and your agreement as to how your information will be managed during and after the study. Please carefully read the points below and tick the appropriate boxes to provide your consent to participate in this study.

[Text from consent form]

Your participation is entirely voluntary, and you can withdraw at any time during the data collection process (one month after data collection starts [exact date to be added here]) and before the data analysis begins (before 1 August 2025). If you choose to withdraw, any answers you have provided will be deleted. Also, when completing the survey, you are free to skip any question you do not wish to answer.

If you have any further questions, please email the main researcher, Heather Duggan, at [hduggan1@sheffield.ac.uk](mailto:hduggan1@sheffield.ac.uk). Thank you in advance for your participation.

[Next button]

**Page 3 onwards:**

**Survey questions**

[Survey questions, with each section starting on a new page, or over several pages for longer sections to break up the questions and make them easier to read.]

**Last Page:**

**Submit your Responses and Thank You**

**By clicking the ‘Submit’ button below, you are consenting to participate in this study, as it is described in the participant information sheet***,* which you can download here:

[Link to downloadable information sheet]

*If you have not yet downloaded a copy of the information sheet for your records, we recommend doing that now.*

**Thank you for completing this survey.**

[Submit button]

Message participants receive after clicking submit:

**Thank you for completing this survey and submitting your responses.**

Thank you for taking the time to share your experiences of delivering CBT-AN-20. Your insights will help us to improve therapists’ and patients’ experiences of this therapy in the future.

**Contact details for further information**

Lead researcher: Heather Duggan ([hduggan1@sheffield.ac.uk](mailto:hduggan1@sheffield.ac.uk)).

Supervisor: Prof. Glenn Waller ([g.waller@sheffield.ac.uk](mailto:g.waller@sheffield.ac.uk)).
