# Supplementary material for: How Do Therapists Experience 20‐Session Cognitive‐Behavioral Therapy for Anorexia Nervosa (CBT‐AN‐20)? A Qualitative Study
Source: Int J Eat Disord. 2026 Jul 28;59(11):2508–20. doi: 10.1002/eat.70174 (PMC13726451; doi:10.1002/eat.70174)
Supplement: Supplementary file 5 [file EAT-59-2508-s005.docx]

**Invitation to Participate Email for CBT-AN-20 Therapist Survey**

The following text will be provided to the clinical leads to send out to all their eligible therapists (current and former employees) to invite them to participate in this survey.

Dear Therapist,

We hope this email finds you well.

We are emailing to invite you to participate in an online Qualtrics survey about your experiences with delivering CBT-AN-20. As a therapist who has experience of delivering CBT-AN-20 to your patients, we highly value your insights. Your survey responses at this early stage of intervention development will help to shape future iterations of this novel therapy and improve patient outcomes.

The survey takes up to 20 minutes to complete. Participation is voluntary and anonymous. Please follow this link for full study details and to complete the survey: [QUALTRICS LINK].

Thank you in advance for your help.

Kind regards,

Heather Duggan (PhD researcher at the University of Sheffield)

Supervisor: Glenn Waller (University of Sheffield).
