# Supplementary material for: How Do Therapists Experience 20‐Session Cognitive‐Behavioral Therapy for Anorexia Nervosa (CBT‐AN‐20)? A Qualitative Study
Source: Int J Eat Disord. 2026 Jul 28;59(11):2508–20. doi: 10.1002/eat.70174 (PMC13726451; doi:10.1002/eat.70174)
Supplement: Supplementary file 6 [file EAT-59-2508-s006.docx]

**Supporting Information G**

**Table G1**

*Comparison of Therapists’ Estimates of Their Confidence in Treatment Effectiveness and Their Ability Before and After Delivering CBT-AN-20*

|  | **Before Delivering CBT-AN-20**  ***M* (SD)** | **After Delivering CBT-AN-20**  ***M* (SD)** | **Mean Difference** | ***t*(df)** | ***p*** |
| --- | --- | --- | --- | --- | --- |
| Therapists’ confidence in treatment effectiveness | 6.10 (1.79) | 7.10 (1.52) | 1.00 | 1.54(9) | .158 |
| Therapists’ confidence in their ability to deliver treatment | 6.00 (2.11) | 7.00 (1.05) | 1.00 | 2.02(9) | .074 |

*Note*. *N* = 10. Data were from a cross-sectional survey and are therefore therapists’ retrospective self-reported estimates of their confidence before and after delivering CBT-AN-20.
