# Supplementary material for: How Do Therapists Experience 20‐Session Cognitive‐Behavioral Therapy for Anorexia Nervosa (CBT‐AN‐20)? A Qualitative Study
Source: Int J Eat Disord. 2026 Jul 28;59(11):2508–20. doi: 10.1002/eat.70174 (PMC13726451; doi:10.1002/eat.70174)
Supplement: Supplementary file 7 [file EAT-59-2508-s007.docx]

**CBT-AN-20 Therapist Survey Questions**

The following questions will form part of a Qualtrics survey to explore therapists’ experiences of CBT-AN-20. The first page will contain an overview of the study and what participation will involve. The second page will contain the information sheet and consent form details. The third page onwards will contain the below survey questions. Each section of survey questions will start on a new page. The last page will allow participants to submit their responses.

**Section A - Expectations of CBT-AN-20**

This section asks four questions about your expectations of CBT-AN-20 before you started to deliver this treatment. Three questions ask you to rate your response on a scale of 0 (not at all) to 10 (completely). Please select the most appropriate option. The last question is open-ended. Please answer in as much detail as appropriate.

**1. How willing did you think patients would be to engage in CBT-AN-20?**

0--------1--------2--------3--------4--------5---------6---------7---------8--------9--------10

not at all	completely	willing	willing

**2. How confident did you feel in the potential effectiveness of CBT-AN-20 for treating patients’ eating disorders?**

0--------1--------2--------3--------4--------5---------6---------7---------8--------9--------10

not at all	completely	confident	confident

**3. How confident did you feel in your ability to deliver CBT-AN-20 competently?**

0--------1--------2--------3--------4--------5---------6---------7---------8--------9--------10

not at all	completely	confident	confident

**4. Please share any comments about your expectations of CBT-AN-20 before delivering this therapy to patients.**

[Open-ended response]

**Section B – Experiences of CBT-AN-20**

This section asks nine open-ended questions and four scale-rating questions about your experiences of delivering CBT-AN-20.

Please provide as much detail as appropriate in your responses to the following nine open-ended questions.

**1. Please describe your overall experience of delivering CBT-AN-20.**

[Open-ended response]

**2. What did you and your patients find most helpful about CBT-AN-20, and why?**

[Open-ended response]

**3. What made CBT-AN-20 difficult for you and your patients, and why?**

[Open-ended response]

**4. Who do you think this therapy is most beneficial for, and why?**

[Open-ended response]

**5. Who do you think this therapy is least beneficial for, and why?**

[Open-ended response]

**6. Please tell us about your experiences of the therapeutic relationship in CBT-AN-20.**

[Open-ended response]

**7. How did your experiences of CBT-AN-20 compare to other therapies you have delivered for patients with anorexia nervosa?** Please name the other therapies in this section. If you have not previously delivered any other therapy for anorexia nervosa, please state N/A.

[Open-ended response]

**8. Please share any comments or suggestions about how CBT-AN-20 might be improved.**

[Open-ended response]

**9. Please share any further comments you have about your experiences of delivering CBT-AN-20.**

[Open-ended response]

The next four questions ask you to rate your response on a scale of 0 (not at all) to 10 (completely). For each question, please select the most appropriate option.

**10. For patients who completed a full course of treatment (in either 20 sessions or faster), how effective was CBT-AN-20?**

0--------1--------2--------3--------4--------5---------6---------7---------8--------9--------10

not at all	completely	effective	effective

**11. How confident did you feel in your ability to deliver CBT-AN-20 competently?**

0--------1--------2--------3--------4--------5---------6---------7---------8--------9--------10

not at all	completely	confident	confident

**12. How likely is it that you would recommend CBT-AN-20 to other therapists?**

0--------1--------2--------3--------4--------5---------6---------7---------8---------9---------10

not at all	Extremely

likely	likely

**13. How likely is it that you would recommend CBT-20-AN to your loved one if they were to need treatment for anorexia nervosa?**

0--------1--------2--------3--------4--------5---------6---------7---------8---------9---------10

not at all	Extremely

likely	likely

**Section C - About you**

We request that you provide your demographic details so that we can explore therapists’ characteristics and how this might relate to their experiences of delivering CBT-AN-20. Responses will be collated to provide an overall summary of participants’ characteristics and will not be identifiable as related to you.

This section of the survey is optional. If you are happy to provide this information, please click the button below to complete six quick additional demographic questions.

[Within an embedded survey]

We recognise and respect that some people prefer not to share certain information about themselves. Please leave blank any questions you do not wish to answer.

**1. How much previous experience do you have in delivering evidence-based therapy for eating disorders (CBT and other therapies)?**

_Years and _Months / Prefer not to say

**2. Job Title/Position**	Clinical Psychologist / CBT Therapist / Assistant Psychologist / Other (please state)/ Prefer not to say

**3. Highest Education Level**	Bachelor’s degree / Masters / PhD / Doctorate in Clinical or Counselling Psychology / Other Counselling or Therapy Qualifications / Other (please state)/ Prefer not to say

**4. Age (in years)**	18 – 24 / 25 – 34 / 35 – 44 / 45 – 54 / 55 – 64 / 65 and above/Prefer not to say

**5. Sex at birth**	Male / Female / Intersex / Prefer not to say

**6. Ethnicity**	Central or East Asian / Southeast Asian / South Asian / Black African / Black Caribbean / Other Black background /

Hispanic or Latin / Middle Eastern or North African / Mixed or multiple ethnicities / White / Other ethnicity (please state)/ Prefer not to say
